# Skipping Meals Is Associated With Symptoms of Anxiety and Depression in U.S. Older Adults

**DOI:** 10.1093/geroni/igaa057.1663

**Published:** 2020-12-16

**Authors:** Loretta Anderson

**Affiliations:** University of Maryland School of Medicine, Reisterstown, Maryland, United States

## Abstract

Previous studies have shown that higher levels of economic hardship in older adults is associated with increased odds of adverse health outcomes such as insomnia, anxiety, and depressive symptoms. The objective of this study was to determine if there was a differential association between individual measures of economic hardship and aforementioned adverse health outcomes. Cross-sectional analysis was conducted using data from the 2013 National Health and Aging Trends Study (NHATS). Logistic models were developed to assess the association between four measures of economic hardship which included not having enough money for food, utility bills, mortgage/rent, or medical bills/prescription drugs. Measures of adverse health outcomes were symptoms of depression, anxiety, and insomnia. There were 4467 community-dwelling older adults (65+) in the analyses. Results indicated those who skipped meals were more likely to have depression, anxiety, and insomnia symptoms than those who did not skip meals. After adjusting for race, age, gender, education, and total number of comorbid health conditions, skipping meals was associated with depression (OR=2.71, p<.05) and anxiety (OR=2.84, p<.01). Skipping meals did not have a statistically significant association with insomnia. The analysis for skipping meals showed a higher odds and more statistically significant results than the other measures of economic hardship listed above. These findings are relevant to population-based efforts to improve quality of life in aging populations and may be of interest to those researchers investigating the gut-brain axis. These findings may also inform future policy efforts to address health disparities and food insecurity in older adults.

